# A Novel Remote Sensing Approach for Prediction of Maize Yield Under Different Conditions of Nitrogen Fertilization

**DOI:** 10.3389/fpls.2016.00666

**Published:** 2016-05-18

**Authors:** Omar Vergara-Díaz, Mainassara A. Zaman-Allah, Benhildah Masuka, Alberto Hornero, Pablo Zarco-Tejada, Boddupalli M. Prasanna, Jill E. Cairns, José L. Araus

**Affiliations:** ^1^Integrative Crop Ecophysiology Group, Plant Physiology Section, Faculty of Biology, University of BarcelonaBarcelona, Spain; ^2^International Maize and Wheat Improvement Center, CIMMYT Southern Africa Regional OfficeHarare, Zimbabwe; ^3^Laboratory for Research Methods in Quantitative Remote Sensing, QuantaLab, Institute for Sustainable Agriculture, National Research CouncilCordoba, Spain

**Keywords:** breeding, crop management, field phenotyping, maize, nitrogen fertilization, NDVI, RGB indices

## Abstract

Maize crop production is constrained worldwide by nitrogen (N) availability and particularly in poor tropical and subtropical soils. The development of affordable high-throughput crop monitoring and phenotyping techniques is key to improving maize cultivation under low-N fertilization. In this study several vegetation indices (VIs) derived from Red-Green-Blue (RGB) digital images at the leaf and canopy levels are proposed as low-cost tools for plant breeding and fertilization management. They were compared with the performance of the normalized difference vegetation index (NDVI) measured at ground level and from an aerial platform, as well as with leaf chlorophyll content (LCC) and other leaf composition and structural parameters at flowering stage. A set of 10 hybrids grown under five different nitrogen regimes and adequate water conditions were tested at the CIMMYT station of Harare (Zimbabwe). Grain yield and leaf N concentration across N fertilization levels were strongly predicted by most of these RGB indices (with *R*^2^~ 0.7), outperforming the prediction power of the NDVI and LCC. RGB indices also outperformed the NDVI when assessing genotypic differences in grain yield and leaf N concentration within a given level of N fertilization. The best predictor of leaf N concentration across the five N regimes was LCC but its performance within N treatments was inefficient. The leaf traits evaluated also seemed inefficient as phenotyping parameters. It is concluded that the adoption of RGB-based phenotyping techniques may significantly contribute to the progress of plant breeding and the appropriate management of fertilization.

## Introduction

Low soil fertility, alongside drought and heat, is a major stress factor limiting crop productivity on a world scale (Stewart et al., 2005). In the case of sub-Saharan Africa, the lack of nitrogen (N) is the main constraint on cereal yields in areas with more than 400 mm average annual rainfall (Buerkert et al., 2001). Therefore, an optimization of N use is critical for increased grain production, especially in the low productive regions. On the other hand, on the basis of environmental and economic sustainability, a more restricted and reasonable use of fertilizers is necessary. Plant scientists, especially breeders and agronomists, face the challenge of solving these limitations while taking into account the additional implications of climate change on food security (Cairns et al., 2012, 2013).

Maize is the second most cultivated cereal worldwide and the most commonly cultivated cereal in Africa in terms of land area and production (FAO, 2013). In particular, agricultural productivity of sub-Saharan Africa remains the lowest in the world partly due to low soil fertility (Cairns et al., 2013; Fischer et al., 2014). Therefore, improving tolerance of maize to low N will increase yields and impact positively on livelihoods and food security (Masuka et al., 2012).

In this sense, two strategies are considered paramount for crop scientists: (i) breeding to improve varieties toward higher nutrient use efficiency and tolerance to nutrient-deficiency (ii) and appropriate fertilization management (Wezel et al., 2014), including precision agriculture (PA; Hatfield, 2000; Chen et al., 2014). Thereby, the implementation of such improvements may increase farmers' profits by maintaining crop yield and reducing the use of resources while preventing further degradation to the environment (Hergert et al., 1996; Delgado et al., 2001; Roberts et al., 2001; Wang et al., 2003). In that sense, technologies for crop monitoring and breeding must be high performing, broad-use and affordable, particularly (but not only) when national agricultural systems, seed companies, or small farmers from developing countries are the targets. Moreover, in the case of breeding, improvements are needed to overcome the field phenotyping bottleneck that limits breeding and advances in PA (Araus et al., 2008; Furbank and Tester, 2011; Araus and Cairns, 2014).

Remote proximal sensing technologies are being used currently for precise management of crops, whereas its potential application for field high throughput phenotyping has gathered increasing interest in recent years (Araus and Cairns, 2014; Liebisch et al., 2015). The classical approach has involved the use of multispectral sensors and the development of numerous vegetation indices associated with vegetation parameters such as above-ground biomass, water and nutrient-deficiency and crop yield (Petropoulos and Kalaitzidisz, 2012). Among the indices, the Normalized Difference Vegetation Index (NDVI) is the most widely used. Concerning crop N performance, several studies have shown that it is possible to quantify it satisfactorily using multispectral data at both the aerial and ground levels (Barnes et al., 2000; Boegh et al., 2002). However, multi and hyper-spectral imagers are relatively expensive and complex from the operational point of view.

As a low-cost alternative, vegetation indices derived from Red-Green-Blue (RGB) cameras have been employed for remote sensing assessment in field conditions, providing a wide-range of phenomic data about genotypic performance under different stress conditions and species, including water stress and foliar diseases in bread wheat, durum wheat and tritordeum and triticale (Casadesus et al., 2007; Casadesús and Villegas, 2014; Vergara-Diaz et al., 2015; Zhou et al., 2015). Moreover, digital sensors have been successfully integrated on board unmanned aerial vehicles (UAV) to assess crop vigor, vegetation coverage, and greenness (White et al., 2012; Andrade-Sanchez et al., 2014; Svensgaard et al., 2014). For example, digital indices derived from RGB images have been proposed for grain yield (GY) assessment in water limiting conditions (Casadesus et al., 2007) and for quantifying leaf N concentration (Rorie et al., 2011). However, the use of RGB images to assess genotypic performance in terms of yield and crop N accumulation in response to different levels of soil fertility has not yet been assessed. RGB images may represent a proper alternative to spectroradiometric approaches at different levels: at the whole trial level from aerial platforms, at the plot level from ground-based measurements or even at the single leaf level replacing leaf chlorophyll meters.

Information derived from plant samples may also be relevant for crop monitoring and phenotyping (Araus and Cairns, 2014). For example the stable isotope composition in plant matter constitutes an integrative selection criterion because it can describe the behavior of the crop under stress (Masuka et al., 2012). Nitrogen isotope composition (δ^15^N) can be employed to characterize the efficiency in using N fertilizers (Evans, 2001; Serret et al., 2008). For its part, implementing carbon isotope composition (δ^13^C) in maize is not clear for assessing genotypic differences due to the C_4_-photosynthetic metabolism of this species, but still appears responsive to differences in growing conditions (Monneveux et al., 2007; Araus et al., 2010). Finally, some other morphological and compositional traits such as the specific leaf area (SLA), N concentration, N per unit leaf area (N/LA), and carbon to nitrogen ratio (C/N), which are in turn related to nitrogen use efficiency, leaf construction, and primary metabolism (Poorter and Evans, 1998; Feng et al., 2008), have the potential to be useful for breeding, but knowledge about their association with crop yield is scarce.

The main goal of this study is to develop affordable easy-to-use new phenotyping tools that increase selection efficiency for grain yield and leaf N concentration under different N fertilization conditions in maize. To accomplish this objective, we compared the accuracy of field-spectroradiometer data vs. RGB-derived vegetation indices assessing GY and leaf N concentration in a set of ten maize hybrids grown in the field under five N-fertilizer levels. Firstly, we assessed the performance of these parameters for all the N-treatments together, and subsequently we dissected the correlations within each N-level for further discussion of phenotyping. Additionally, simple regression models were made for GY prediction and these models were tested and validated against the experimental yield of another trial. The performance of the leaf parameters N/LA, C/N, SLA, and δ^13^C and δ^15^N were also studied with the aim of relating these structural and compositional leaf properties with crop performance and phenotyping data. All RGB and UAV imagery were obtained at flowering stage in order to integrate the differences in crop performance from plant emergence to flowering stage, when the number of kernels per ear is determined.

## Materials and methods

### Experimental design and growing conditions

Field trials were carried out at the Southern Africa regional station of CIMMYT (International Maize and Wheat Improvement Center) located in Harare (17°43′32″S, 31°00′59″E) where two field experiments were studied. Before sowing, soil pH, total soluble salts (TSS), nitrogen as nitrate (${\text{NO}}_{3}^{-}$) and phosphorus (P_2_O_5_) were analyzed in three soil depth ranges (0–30, 30–60, and 60–90 cm) and six replicates for each depth range were produced. Mean values for the full soil profile were pH = 5.8, TSS = 240.9 ppm, ${\text{NO}}_{3}^{-}=4.12$ ppm, and P_2_O_5_ = 18.93 ppm.

Ten maize hybrids were sown, three of them were commercial hybrids (PAN7M-81, SC635, SC537) and the other seven were maize hybrids developed at CIMMYT (TH11894, TH127591, TH127053, TH127618, TH13466, CZHH1155, TH127004). These maize hybrids cover a big range of agronomical sensitivity to low nitrogen conditions. A split-plot arrangement in a randomized block design was set up and five nitrogen fertilization levels (0, 10, 20, 80, and 160 kg·ha^−1^ NH_4_NO_3_) were applied in both trials. Two and three replicates were set for the first and second trials, with 100 and 150 being the respective number of plots in each trial (trials S and P, respectively). A two-row border was sown between fertilization treatments and on the edges of the trial to prevent spatial variability.

Seeds were sown during the wet season, on December 23th 2013, in two rows per plot; rows were 4 m long and 75 cm apart (6 m^2^/plot), with 17 planting points per row and 25 cm between plants within a row. All trials were homogeneously fertilized with 400 kg·ha^−1^ of super-phosphate and potassium oxide fertilizer (P_2_O_5_ 14% and K_2_O 7%). Weather conditions throughout growing season were recorded with a weather station. The mean temperature was 18.9°C, mean humidity 81.2 and total rainfall during the crop period was 563.1 mm, therefore, preventing the water deficit in these rainfed conditions.

The trials were harvested on May 20th 2014. The central 3.5 m of each row was harvested discarding 2 plants at each end, thus the collected weight corresponded to 5.25 m^2^ (0.75 m apart × 2 rows × 3.5 m long). The cobs were threshed and the grains dried until they reached around 12% moisture, and then the grain from each plot was weighed. Grain yield (GY, Mg·ha^−1^) was calculated as follows: (X kg plot^−1^ × 10)/5.25 where X is the grain weight per plot.

### NDVI calculation

**Figure 1:**
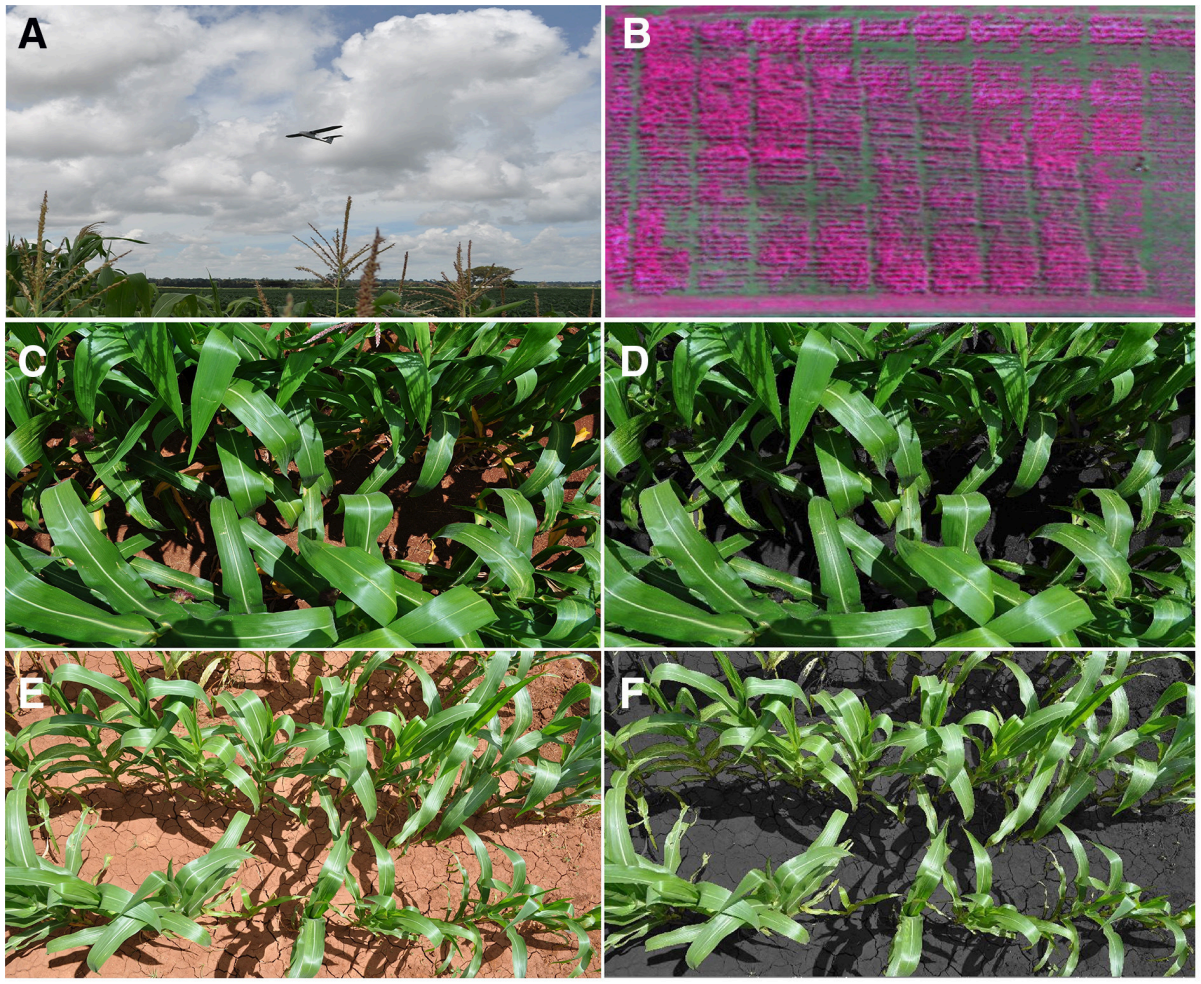
**(A)** The unmanned aerial vehicle flying over the maize crops; **(B)** Multispectral false-color image at the aerial level showing near infrared (800 nm) as red, green (550 nm) as blue and red (670 nm) as green, spatial resolution of 10 cm/px; **(C)** RGB digital image from the high-nitrogen fertilization treatment at the canopy level; **(D)** and its resulting processed image with BreedPix; **(E)** RGB image from the low-nitrogen treatment and **(F)** its respective processed image.

The normalized difference vegetation index (NDVI) was calculated using the equation:

$$\begin{array}{l} NDVI\;=\;\left(NIR\;-\;R\right)∕\left(NIR\;+\;R\right) \end{array}$$

where *R* is the reflectance in the red band and NIR is the reflectance in the near-infrared band. NDVI was obtained around the flowering stage by using two different approaches: using ground measurements and from aerial multispectral images (Figure 1).

The NDVI of individual plots at ground level (NDVI_ground_) was determined with a ground-based portable spectroradiometer with an active sensor (GreenSeeker handheld crop sensor, Trimble, USA). This equipment uses the spectral wavelengths 650–670 nm as the red band and 765–795 nm as the near infrared. The distance between the sensor and the plots was kept constant using a ladder, around 0.5–0.6 m above and perpendicular to the canopy. The whole areas of the two trials were measured from 12 to 14 h on March 3rd and 4th, 2014.

The aerial NDVI index (NDVI_aerial_) was obtained using a UAV-based remote sensing platform developed by Airelectronics (Montegancedo campus, Spain) in collaboration with the Crop Breeding Institute-Zimbabwe, CIMMYT, QuantaLab at the Institute for Sustainable Agriculture (IAS-CSIC, Spain) and the University of Barcelona. This aerial platform was equipped with a multispectral camera (ADC-Lite, Tetracam, Inc., Chatsworth, CA, US), which provides spectral images on the green, the red and the near-infrared bands, with a final ground resolution of 10 cm per pixel when flying at an object distance of 150 m. These bands are approximately equal to the Landsat Thematic Mapper (TM) bands TM2, TM3, and TM4, respectively, so that the spectral wavelengths from 630 to 690 nm represent the red band and 760 to 920 nm the near infrared band. The flight was conducted at an altitude of 150 m at midday on a sunny day when crops were around the flowering stage. The collected images covered 220 out of the total 250 plots, completely covering the block S trial (100 plots) and partially covering block P (120 of the total of 150 plots). Aerial images were subsequently corrected and calibrated with ImapQ (QuantaLab-IAS-CSIC, Cordoba, Spain) which converts images to radiance. Mosaicking and rectifying processes were applied with Autopano (Kolor SARL, Francin, France) by applying the image stitching technique (SIFT algorithm) in addition to a manual orthorectification from several checkpoints selected. NDVI values were finally extracted from the images using ENVI software (Exelis Visual Information Solutions, Boulder, Colorado, USA).

### RGB indices

Vegetation indices derived from red-green-blue (RGB) images were evaluated at the plot and the single leaf level (RGB_canopy_ and RGB_leaf_ indices, respectively; Figure 1). In the case of RGB_canopy_, one digital RGB picture was taken per plot by holding the camera about 0.8–1.0 m above the canopy, in a zenithal plane and focusing near the center of each plot. Plot images were taken on the same days as the measurements with the ground spectroradiometer using a Nikon COOLPIX S8000 digital compact camera without flash and with a focal length of 54 mm and were saved in a 4288 × 2848 pixel JPEG format. Later, six leaves per plot were taken from the S trial (100 plots) and were subsequently scanned with a Dell 2155 cdn multifunction color printer (Round Rock, TX, USA). Finally, scanned images were saved in the same format with a resolution of 2338 × 1653 pixels and RGB_leaf_ indices calculated as below.

Subsequently, images were analyzed with the open source Breedpix 0.2 software (Casadesus et al., 2007) designed to process digital images. This software enables calculation of several RGB vegetation indices based on the different properties of color inherent in RGB images. RGB VIs were obtained either from the average color of the whole image or from the hue histogram in each image. BreedPix produces several automatic conversions of the original RGB image to other color spaces (i.e., each model that numerically represents the color in terms of different coordinates). Four VIs (a^*^, b^*^, u^*^, and v^*^) belonging to CIE (from the French abbreviation of International Commission on Illumination) color spaces were calculated and used in this study. The software require the use of Java Advanced Imaging (JAI) for the conversion of RGB color space to CIE-XYZ color space and the resulting coordinates are subsequently converted to other color spaces. First, the VIs a^*^ and b^*^ belong to CIE-Lab color space, being L^*^ the lightness dimension and a^*^ and b^*^ the color-opponent coordinates. Red/green opponent-colors are represented along a^*^ axis, whereas b^*^ axis represent the yellow/blue opponent colors. Similarly, u^*^ and v^*^ indices represent the axis in the chromaticity diagram of CIE-Luv color space. Thereby the software obtains the average values of these components of color for each one of the processed images. Hue component is calculated using the JAI functions which employ the formulae described in Seul et al. (2000) whereas the components of CIE-Lab and CIE-Luv color spaces are calculated as described in Trussell et al. (2005). The relative green area (GA) and the relative “greener area” (GGA) are based on the sum of frequencies of the histogram classes included in a certain range of hue in the image. GA is the percentage of pixels in the image in the hue range from 60 to 180°, that is, from yellow to bluish green. On the other hand GGA is somewhat more restrictive since the range of hue considered by this index is from 80 to 180°, excluding yellowish-green tones and therefore, it more accurately describes the amount of photosynthetically active biomass and leaf senescence.

### Analysis of leaf parameters

The leaf portions in the RGB_leaf_ indices were also used the subsequent measures. Firstly, immediately before being scanned, a handheld spectroradiometer developed for leaf chlorophyll measurements (Minolta SPAD-502, Spectrum Technologies Inc, Plainfield, IL, USA) was used to measure the index related to leaf chlorophyll content (LCC). Four measurements were made for each leaf segment. Secondly, the leaves were oven dried at 70°C for 24 h and the dry weight was measured. Then the specific leaf area (SLA) was calculated using the equation

$$\begin{array}{l} SLA\;=\;LA\;∕\;DW \end{array}$$

where *LA* is the total leaf area (m^2^) measured previously from the scanned images using the open-source Java-based software ImageJ (http://rsb.info.nih.gov.sire.ub.edu/ij/) and DW is the corresponding dry weight (kg).

Finally, dry leaves were ground to a fine powder and 0.7–0.9 mg of leaf dry matter from each plot was weighed and sealed into tin capsules. Stable carbon (^13^C/^12^C) and nitrogen (^15^N/^14^N) isotope ratios as well as the leaf N and C concentrations (%) were measured using an elemental analyser (Flash 1112 EA; Thermo Finnigan, Bremen, Germany) coupled with an isotope ratio mass spectrometer (Delta C IRMS, Thermo Finnigan) operating in a continuous flow mode. Samples were loaded into a sampler and analyzed. Measurements were conducted at the Scientific Facilities of the University of Barcelona. Isotopic values were expressed as a composition notation (δ) as follow:

$$\delta \;(‰)\;=\;{{(}^{13}C{/}^{12}C)}_{sample}/\;{(}^{13}C{/}^{12}C){\;}_{standard}-1$$

where “sample” refers to plant material and “standard” to international secondary standards of known ^13^C/^12^C ratios (IAEA CH7 polyethylene foil, IAEA CH6 sucrose and USGS 40 L-glutamic acid) calibrated against Vienna Pee Dee Belemnite calcium carbonate with an analytical precision (standard deviation) of 0.15%0. The same δ notation was used for the ^15^N/^14^N ratio expression but with the standard referring to air. For nitrogen, international isotope secondary standards IAEA N1, IAEA N2, IAEA NO3, and USGS 40 were used with a precision of 0.3%0. Further, the C/N ratio was obtained from these analyses and total nitrogen concentration per unit leaf area (N/LA) was calculated with the formula:

$$\begin{array}{l} N\;∕LA\;=\;\left(\frac{DW}{LA}\right)x\;N \end{array}$$

where *LA* is the total leaf area (m^2^), DW is the corresponding dry weight (g) and *N* is its nitrogen concentration (in % dry matter).

### Statistical analysis

Statistical analyses were conducted using SPSS 21 (IBM SPSS Statistics 21, Inc., Chicago, IL, USA). Multiple variance analyses, the multiple comparison Duncan *post-hoc* test and bivariate correlations were performed. The presented leaf parameters (LCC, N, δ^15^N, N/LA, SLA, δ^13^C, C/N) and RGB_leaf_ indices from scanned leaves were only analyzed for the S trial, whereas the NDVI_ground_, NDVI_aerial_, and RGB_canopy_ indices were studied for both trials. The determination coefficients of the linear relationships of GY and leaf N concentration with the vegetation indices NDVI and RGB were calculated for the entire trials and within each N fertilization treatment. All graphs were performed with SigmaPlot 10.0 (Systat Software Inc., San Jose, California, US).

## Results

Significant differences in GY between genotypes and nitrogen-fertilization levels were observed in this study (Table 1) with GY increasing in response to N fertilization (Table 2). Differences within nitrogen-input levels were also detected with both (ground and aerial) NDVI approaches and with all RGB_canopy_ indices except by a^*^. Genotypic differences were detected by the RGB_canopy_ indices GGA, GA, a^*^, u^*^, and hue, whereas among the spectroradiometric indices only the NDVI at the ground level detected genotypic differences.

**Table 1:** *****P***-values from multivariate analysis of variance with two fixed factors: genotype and nitrogen level and its interaction (GxN)**.

			**Genotype**	**Nitrogen level**	**G × N**
GY			< 0.001	< 0.001	0.884
**SPECTRAL INDICES**					
		NDVI_aerial_	0.509	< 0.001	0.745
		NDVI_ground_	< 0.001	< 0.001	0.500
**RGB**_*canopy*_ **INDICES**					
		hue	0.032	< 0.001	0.997
		a^*^	0.006	0.125	0.727
		b^*^	0.590	< 0.001	0.997
		u^*^	0.012	< 0.001	0.801
		v^*^	0.567	< 0.001	0.993
		GA	0.002	< 0.001	0.708
		GGA	< 0.001	< 0.001	0.564
**RGB**_*leaf*_ **INDICES**					
		hue	0.412	< 0.001	0.817
		a^*^	0.364	< 0.001	0.882
		b^*^	0.580	< 0.001	0.999
		u^*^	0.310	< 0.001	0.687
		v^*^	0.787	< 0.001	0.999
		GA	0.042	< 0.001	0.604
		GGA	0.289	< 0.001	0.225
LCC			0.939	< 0.001	0.973
**ANALYZED PARAMETERS**					
		Leaf %N	0.026	< 0.001	0.999
		N/LA	0.347	< 0.001	0.972
		C/N	0.120	< 0.001	1.000
		δ^15^N	0.375	< 0.001	1.000
		δ^13^C	< 0.001	< 0.001	0.937
		SLA	0.822	0.004	0.89

*As dependent variables, grain yield (GY), leaf nitrogen concentration (%N), NDVI at aerial and ground levels, RGB indices from canopy images and scanned leaves, leaf chlorophyll content (LCC), nitrogen per unit area (N/area), the stable carbon (δ^13^C), and nitrogen (δ^15^N) isotope composition, carbon nitrogen ratio (C/N) and specific leaf area (SLA)*.

**Table 2:** **Means of grain yield (GY) (Mg·ha^**−1**^) from the two trials, leaf nitrogen concentration (%N), nitrogen per unit leaf area (N/LA), specific leaf area (SLA), the stable carbon (δ^**13**^C), and nitrogen (δ^**15**^N) isotope composition and the leaf C/N ratio according to the ten hybrids and the five nitrogen levels**.

	**GY (Mg·ha^−1^)**	**%N**	**N/LA (g/m^2^)**	**SLA (m^2^·kg)**	**C/N ratio**	**δ^15^N (%0)**	**δ^13^C (%0)**	**LCC (u)**
**GENOTYPE**								
PAN 7M-81	5.03 b	2.203 ab	111.24 ab	20.23 a	21.07 b	4.53 b	–11.7 ab	41.53 a
TH11894	5.12 b	2.196 ab	101.75 a	21.48 a	20.77 b	3.78 ab	–11.49 cd	42.02 a
TH127591	5.14 b	2.312 abc	117.87 ab	20.41 a	19.75 ab	4.04 ab	–11.79 a	43.06 a
TH127053	5.7 bc	2.32 abc	118.29 ab	19.56 a	19.75 ab	4.18 ab	–11.55 bcd	41.13 a
SC635	3.52 a	2.506 bc	125.31 ab	19.98 a	18.38 ab	3.66 ab	–11.77 a	41.84 a
TH127618	5.38 b	2.621 c	130.12 ab	20.54 a	17.27 a	3.64 ab	–11.63 abc	43.56 a
TH13466	5.75 bc	2.4 abc	117.62 ab	20.91 a	18.96 ab	3.76 ab	–11.45 d	42.57 a
CZH1155	4.63 b	2.526 bc	133.84 b	18.9 a	18.48 ab	3.19 a	–11.64 abc	42.28 a
TH127004	6.49 c	2.138 a	113.71 ab	19.05 a	21.35 b	3.87 ab	–11.71 ab	41.76 a
SC537	4.67 b	2.38 abc	121.71 ab	20.32 a	19.65 ab	3.79 ab	–11.7 ab	42.39 a
**NITROGEN LEVEL**								
0	3.13 a	1.887 a	107.62 a	17.82 a	23.75 d	5.612 e	–11.584 bc	32.5 a
10	3.9 b	1.881 a	92.78 a	20.6 b	23.43 d	4.613 d	–11.59 bc	33.4 a
20	4.61 c	2.089 a	107.09 a	19.91 ab	21.01 c	3.877 c	–11.512 cd	40.4 b
80	6.48 d	2.739 b	138.68 b	20.14 b	16 b	2.898 b	–11.652 b	50.9 c
160	7.59 e	3.206 c	149.56 b	22.22 b	13.54 a	2.218 a	–11.869 a	53.8 d

*Letters are significantly different according to Duncan's multiple range test (P < 0.05)*.

Leaf N concentration varied significantly between genotypes and the effect of N-fertilization levels was highly significant (Table 1) with values increasing as N fertilization increased (Table 2). All the RGB_leaf_ indices detected very significant differences between nitrogen treatments and genotypic differences were also found with GA (Table 1). At the same time, LCC also indicated highly significant differences between N fertilization levels but not genotypic differences.

All the analyzed leaf parameters (N, N/LA, SLA, δ^15^N, δ^13^C, C/N) were highly sensitive to variations in N fertilizer levels (Table 1). In contrast, apart from leaf N concentration, genotypic differences were only detected for δ^13^C. Increasing N fertilization caused significant increases in leaf N, N/LA and SLA while δ^15^N and the C/N ratio decreased (Table 2). Additionally, differences among N fertilization levels were also found in δ^13^C but its trend was somewhat different: in the low-N levels δ^13^C was quite steady and then it decreased at 80–160N, whereas leaf-N concentration increased.

Additionally, the effect of changing light in outdoor conditions was evaluated in RGB indices obtained from canopy images (Table S1). For this purpose, 57 plots were photographed twice in nearly consecutive days, firstly in a sunny day and secondly in a partly cloudy day. All indices were strongly correlated between replicates (*p* < 0.001), particularly the indices GA, GGA, u^*^, a^*^ (*R*^2^ > 0.72).

### Grain yield assessment across nitrogen regimes and genotypes

**Figure 2:**
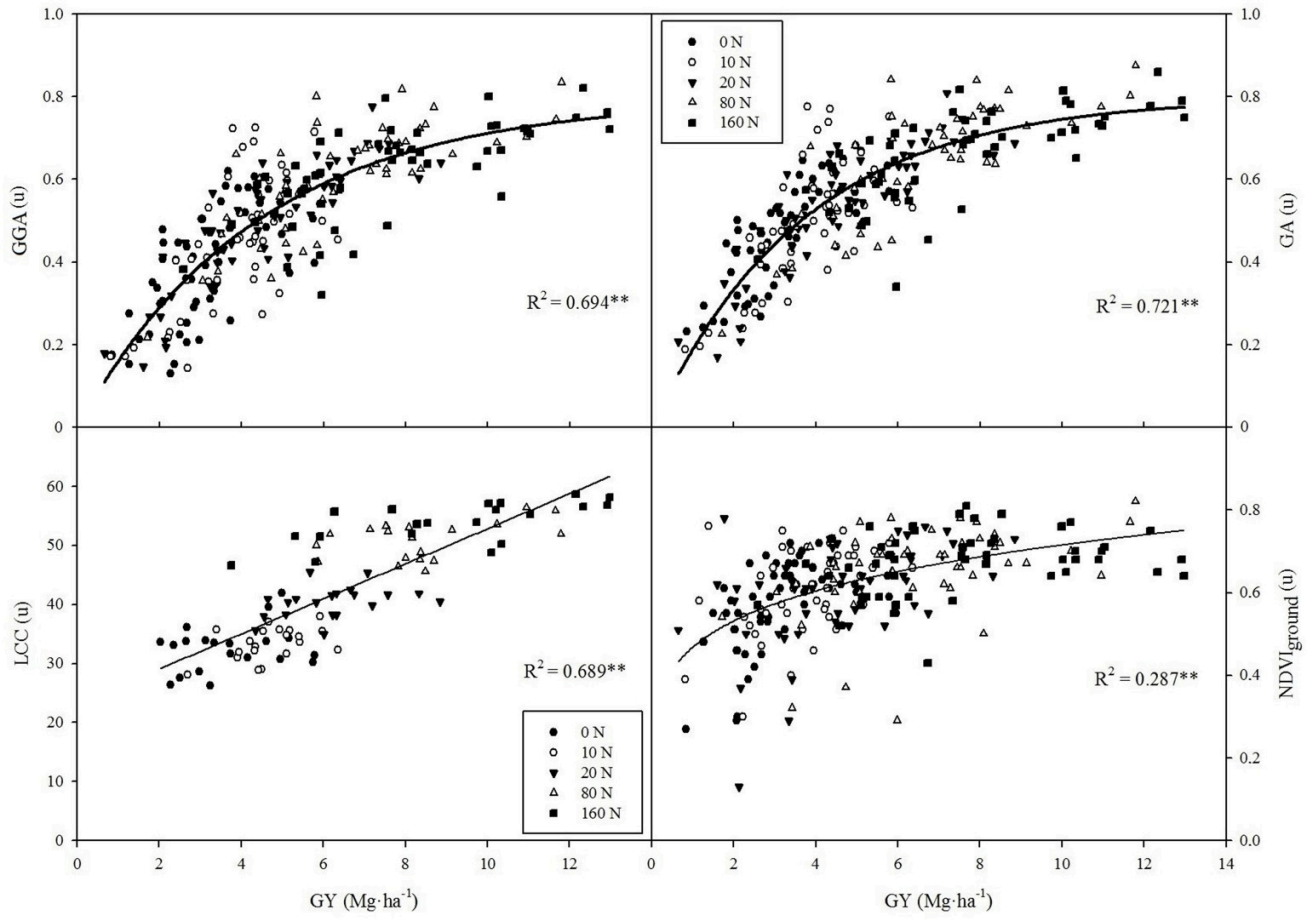
**Correlations between grain yield (GY) and leaf chlorophyll content (LCC), the RGB indices GA and GGA at the canopy level and NDVI at the ground level**. ***R***^**2**^, determination coefficient; ^**^P < 0.001.

All vegetation indices (either ground and aerial NDVI, RGB_canopy_, or LCC) were strongly correlated with GY variation across the whole set of plots of the two trials. The best results were obtained by using the RGB-indices GA and GGA at the canopy level, which showed an exponential regression model and explained 70–72% of GY variability (Figure 2). Meanwhile, the RGB_canopy_ indices u^*^ and a^*^ evolved inversely with increasing GY and demonstrated lower accuracy (*R*^2^ = 0.326 and *R*^2^ = 0.302, respectively, data not shown). In contrast, LCC evolved linearly with increases in GY and explained 69% of GY variation (Figure 2). Finally, both NDVI approaches followed a power regression model and their determination coefficients were moderate and similar (NDVI_ground_ at Figure 2; NDVI_aerial_ *R*^2^ = 0.293, data not shown).

Additionally, simple regression models from the P trial that explained GY across the different N fertilization levels were obtained by using the different VIs and validated for their accuracy in estimating the GY of the S trial (Table 3). The estimated GY from all VIs always fitted satisfactorily with the experimental GY for the entire trial. The determination coefficients increased further when six hybrids contrasting in their grain yield were selected, with three of them being high-yielding and the remaining ones low-yielding. Genotypic differences were found in the estimated GY from GGA, NDVI_aerial_ and NDVI_ground_ between the six selected hybrids and the experimental GY was also significantly different. Moreover, in all models, differences between N fertilization levels were always detected by the estimated GY.

**Table 3:** **Simple regression models obtained with different Vegetation Indices (the spectroradiometric indices NDVI_***aerial***_, NDVI_***ground***_, and the RGB_***canopy***_ indices GA and GGA) in the P trial, explaining Grain Yield (GY) variation across nitrogen fertilization levels, were used for GY estimation in the S trial**.

	**GY exp. vs. GY est**.					
	**Predictors**	**Simple Regression models**	***R*^2^ for all trial**	***R*^2^ for 6 hybrids**	**Genotype**	**N-level**
GY est.	GA	GY = e ^((GA−0.195)∕0.24)^	0.674^**^	0.711^**^	0.054	<0.001
	GGA	GY = e ^((GGA−0.151)∕0.248)^	0.684^**^	0.719^**^	0.040	<0.001
	NDVI_aerial_	GY = e ^((NDVIaerial−0.192)∕0.094)^	0.452^**^	0.543^**^	0.002	<0.001
	NDVI_ground_	GY = e ^((NDVIground−0.461)∕0.109)^	0.231^**^	0.324^**^	0.001	<0.001
GY exp.		GY 3-3 hybrids	–	–	0.044	<0.001

*The fit of the estimated Grain Yield (GY est.) to the experimental Grain Yield (GY exp.) was tested with the determination coefficient (R^2^) for the entire trial and for six yield-contrasting hybrids. P-values were analyzed for all estimated GYs and for the experimental GY using the six selected hybrids*.

** *P < 0.001*.

### Grain yield assessment across genotypes within each N regime

To further assess the accuracy of these indices, the determination coefficients for GY prediction within each N-input level across genotypic means were performed (Table 4). GGA, GA, u^*^, and a^*^ indices were correlated significantly to GY variation within all N levels, whereas both NDVI approaches were correlated significantly to GY only for some of the studied N levels. By contrast, LCC did not correlate with GY across plots within any of the N levels.

**Table 4:** **Determination coefficients (***R***^**2**^) of RGB-indices from canopy images (RGB_***canopy***_), aerial NDVI, ground NDVI, leaf chlorophyll content (LCC), the leaf nitrogen concentration on a dry matter basis (Leaf %N), the nitrogen concentration on a leaf area basis (N/LA), the ratio of carbon to nitrogen concentration (C/N), the stable carbon (δ^**13**^C) and nitrogen (δ^**15**^N) isotope composition and the specific leaf area (SLA) predicting grain yield in the five N levels separately (0, 10, 20, 80, and 160 kg·ha^**−1**^ NH_**4**_NO_**3**_) following linear regression models**.

	**0N**	**10N**	**20N**	**80N**	**160N**
**SPECTRAL INDICES**					
NDVI_aerial_	0.019 ns	0.415^*^	0.092 ns	0.074 ns	0.621^**^
NDVI_ground_	0.741^**^	0.381 ns	0.421^*^	0.212 ns	0.289 ns
**RGB**_*canopy*_ **INDICES**					
hue	0.632^**^	0.653^**^	0.717^**^	0.364 ns	0.729^**^
a^*^	0.706^**^	0.634^**^	0.627^**^	0.524^*^	0.464^*^
b^*^	0.040 ns	0.160 ns	0.046 ns	0.056 ns	0.889^**^
u^*^	0.709^**^	0.608^**^	0.666^**^	0.491^*^	0.569^*^
v^*^	0.079 ns	0.239 ns	0.001 ns	0.120 ns	0.798 ^**^
GA	0.771^**^	0.659^**^	0.704^**^	0.501^**^	0.764^**^
GGA	0.872^**^	0.664^**^	0.774^**^	0.555^*^	0.748^**^
LCC	0.148 ns	0.163 ns	0.076 ns	0.021 ns	0.004 ns
Leaf %N	0.059 ns	0.100 ns	0.014 ns	0.001 ns	0.504^*^
N/LA	0.037 ns	0.046 ns	0.006 ns	0.014 ns	0.189 ns
C/N	0.085 ns	0.069 ns	0.006 ns	0.009 ns	0.365 ns
δ^13^C	0.002 ns	0.353 ns	0.019 ns	0.073 ns	0.001 ns
δ^15^N	<0.001 ns	0.007 ns	0.005 ns	0.025 ns	0.065 ns
SLA	0.004 ns	0.002 ns	0.026 ns	0.008 ns	0.030 ns

* P < 0.05;

** *P < 0.001; ns, non-significant*.

### Leaf nitrogen assessment across N regimes and genotypes

**Figure 3:**
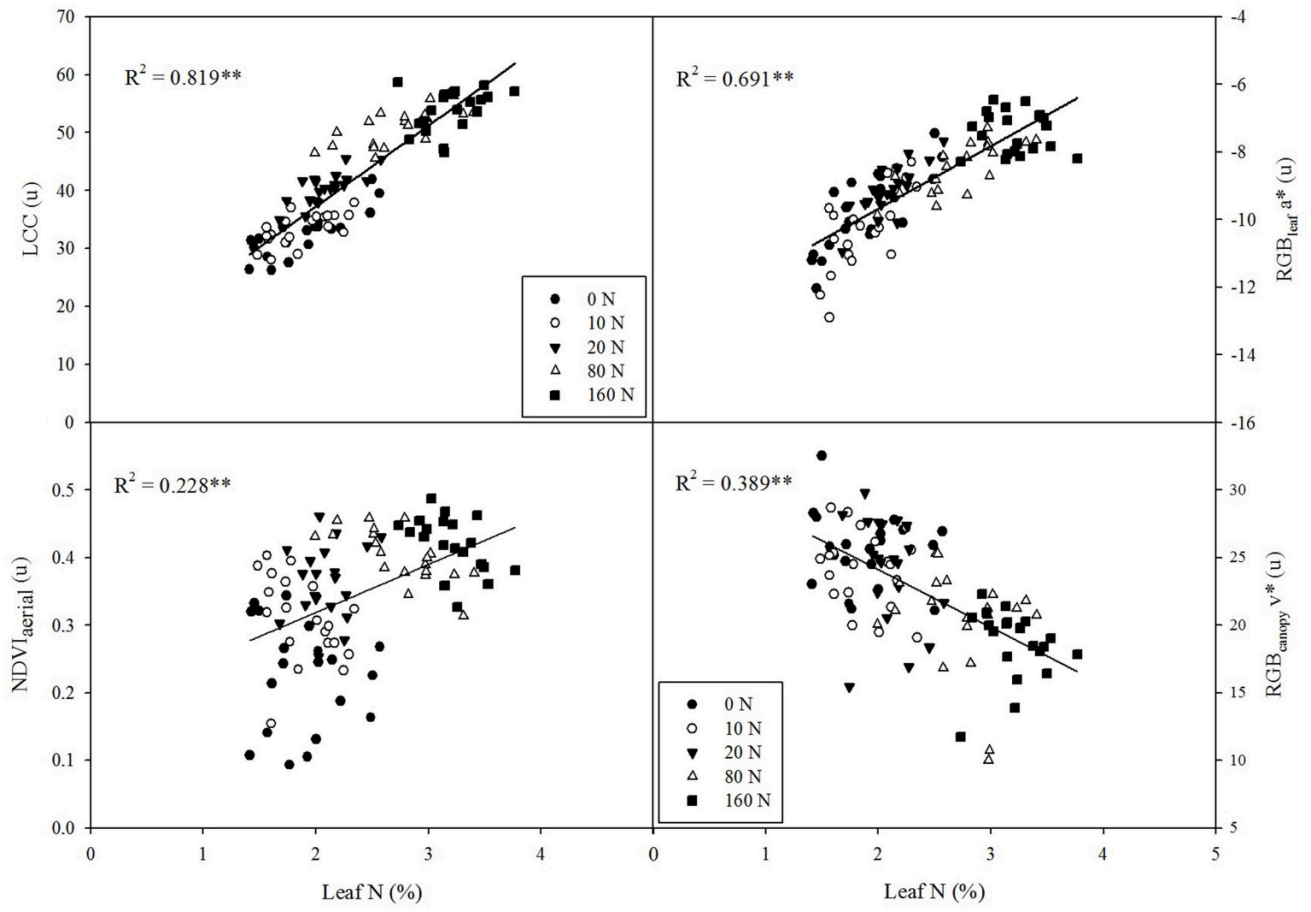
Correlations between leaf nitrogen concentration (%N) and leaf chlorophyll content (LCC), the RGB index a* at the leaf level, the NDVI at the ground level, and the RGB index v^*^ at the canopy level. *R*^2^, determination coefficient; ^**^P < 0.001.

LCC was the best predictor of leaf N concentration across the entire trial, explaining more than 80% of N variability, moderately surpassing the fitting accuracy of the RGB_leaf_ indices (Figure 3). Thus, the RGB_leaf_ index a^*^ explained about 69% of leaf N variation across N fertilization treatments (Figure 3) and u^*^, b^*^, and v^*^ were quite similar (*R*^2^ = 0.682, *R*^2^ = 0.643, and *R*^2^ = 0.621, respectively, data not shown). For its part, NDVI_aerial_ was also a good predictor of leaf N (Figure 3), whereas NDVI_ground_ was less accurate in its prediction (*R*^2^ = 0.116, data not shown). Finally, the RGB index v^*^ at the canopy level was more related to leaf N than it was to GY, and it was shown to be a reasonably good predictor of leaf N across the whole trial (Figure 3).

### Leaf nitrogen assessment across genotypes within each N regime

A table depicting the determination coefficient between the RGB_leaf_ indices, NDVI_aerial_, NDVI_ground_, and LCC against leaf N across genotypic means within each of the N fertilization levels is presented (Table 5). In the low-N treatments (0N to 20N) the best determination coefficients were provided by the RGB_leaf_ indices b^*^, v^*^, u^*^, and a^*^. In addition, most of the RGB_leaf_ indices were also sensitive to leaf N variation at the 80N level but none of them related significantly at the 160N level. For its part, LCC showed a quite similar accuracy compared with the RGB_leaf_ indices in their predictions of leaf N within the low-N levels, but it was not significantly correlated in the high-N fertilization levels (Table 5). Finally, NDVI_aerial_ was especially sensitive to leaf N variations in the high-N and 0N treatments, whereas NDVI_ground_ was generally unrelated to leaf N within each N treatment.

**Table 5:** **Determination coefficients (***R***^**2**^) of RGB-indices from scanned leaves (RGB_***leaf***_), leaf chlorophyll content (LCC), ground NDVI, aerial NDVI, the nitrogen concentration on a leaf area basis (N/LA), the ratio of carbon to nitrogen (C/N), leaf stable carbon (δ^**13**^C) and nitrogen (δ^**15**^N) isotopic composition and specific leaf area (SLA) predicting leaf nitrogen concentration on a dry matter basis separately in the five N fertilization levels (0, 10, 20, 80, and 160 kg·ha^**−1**^ NH_**4**_**NO**_**3**_)**.

	**0N**	**10N**	**20N**	**80N**	**160N**
**SPECTRAL INDICES**					
NDVI_aerial_	0.545^*^	0.242 ns	0.352 ns	0.755^**^	0.650^**^
NDVI_ground_	0.113 ns	0.223 ns	0.285 ns	0.403^*^	0.057 ns
**RGB**_*leaf*_ **INDICES**					
hue	0.081 ns	0.155 ns	0.188 ns	0.094 ns	0.012 ns
a^*^	0.588^*^	0.261 ns	0.567^*^	0.500^*^	0.149 ns
b^*^	0.526^*^	0.489^*^	0.566^*^	0.437^*^	0.265 ns
u^*^	0.607^**^	0.171 ns	0.534^*^	0.507^*^	0.073 ns
v^*^	0.581^*^	0.546^*^	0.429^*^	0.316 ns	0.192 ns
GA	0.097 ns	0.013 ns	0.185 ns	0.517^*^	0.222 ns
GGA	0.261 ns	0.026 ns	0.229 ns	0.359 ns	0.026 ns
**LEAF PARAMETERS**					
LCC	0.587^*^	0.426^*^	0.401^*^	0.168 ns	0.005 ns
N/LA	0.810^**^	0.127 ns	0.643^**^	0.496^**^	0.552^**^
C/N	0.973^**^	0.947^**^	0.918^**^	0.980^**^	0.939^**^
δ^13^C	0.318 ns	0.083 ns	0.069 ns	0.158 ns	0.073 ns
δ^15^N	0.006 ns	0.004 ns	0.167 ns	0.183 ns	0.295 ns
SLA	0.167 ns	0.069 ns	0.219 ns	0.022 ns	0.121 ns

* P < 0.05;

** *P < 0.001; ns, non-significant*.

### Leaf parameters performance and relationships with VIs and yield

Leaf N was strongly negatively correlated across N levels with δ^15^N and the C/N ratio and to a lesser extent with δ^13^C and SLA (Table S2). Correlations of these traits with GY were also negative but weaker, except for SLA which did not correlate.

Most of the RGB indices (both at the leaf and canopy scales), the LCC and the NDVI correlated with N/LA across N regimes, but always more moderately than they correlated with leaf N concentration (Table S2). The association of δ^15^N with NDVI, LCC, and RGB indices (at the both scales) was highly significant and in some cases their correlation coefficients were higher than the respective coefficients between δ^15^N and leaf N. Similarly, δ^13^C was fairly well correlated with most of the RGB indices (especially at the leaf scale) and LCC. Regarding the C/N ratio, LCC was the best predictor but this correlation was smaller than with leaf N concentration. However, most of the RGB_leaf_ indices (a^*^, b^*^ u^*^, v^*^, GA), the RGB_canopy_ indices (hue, u^*^, GA, GGA) as well as NDVI_ground_ and NDVI_aerial_ correlated more strongly with the leaf C/N ratio than they did with leaf N (Table S1). Finally, SLA correlated strongly with the RGB_leaf_ indices GA and GGA, and slightly with both NDVIs.

The relationships between leaf N, N/LA, C/N, δ^13^C, δ^15^N, and SLA with GY across genotypes within N fertilization treatments were almost all non-significant except for leaf N in the 160N treatment (Table 4). Regarding the genotypic correlations within each N fertilization level of these leaf traits with leaf N, only the leaf N derived parameters (C/N and N/LA) were significantly correlated (Table 5).

## Discussion

### Crop monitoring and phenotyping parameters for GY estimation

As previously found in other studies in wheat grown under different stress conditions (Casadesus et al., 2007; Morgounov et al., 2014; Vergara-Diaz et al., 2015), the RGB_canopy_ indices (from BreedPix software) measured at flowering were strongly correlated with GY. RGB-based indices may perform far better than NDVI for GY prediction, which has been recently described under water and biotic stresses in wheat (Elazab et al., 2015; Vergara-Diaz et al., 2015; Zhou et al., 2015). The lower accuracy of NDVI in comparison to digital-based RGB indices can be explained in several ways. On the one hand, graphs clearly highlight (Figure 2) that the variability in the canopy NDVIvalues at ground level is small, with more than 90% of values being in the range 0.5–0.8 and with the NDVI values in the low N treatments being already relatively high (e.g., average of NDVI_ground_ = 0.57 in the 0N treatment). Therefore, the NDVI values remained almost unchanged as GY increased from 4 to 13 Mg ha^−1^. These results support the previously reported saturation of reflectance spectra in the red and near-infrared regions, such that increasing leaf area does not involve a parallel increase in NDVI values (Hobbs, 1995; Elazab et al., 2015). Thus, the relationship between NDVI and aerial biomass saturates as canopies become denser (i.e., LAI > 4) and as a consequence the relationship between the NDVI and GY also worsened as GY increased. Moreover near-infrared reflectance is sensitive to canopy architecture variations (Gitelson et al., 2002) which surely affected NDVI measurements in maize canopies. The use of multi-angular spectral data may solve these problems by capturing the scattering of sunlight by vegetation, which enables to assess three-dimensional vegetation structures (Hasegawa et al., 2010). Whereas this approach may improve the estimation of NDVI (and other spectral indices) for phenotyping, the increasing complexity (i.e., more time and resources needed) of the method makes it less feasible as low-cost alternative.

For its part, the range of variability in the RGB_canopy_ index, GA, was much wider (only 63% of values were in the range of 0.5–0.8) and GA values in the low N treatments were somewhat smaller (average GA = 0.46 in the 0N treatment) than those of the NDVI, and in fact GY correlated much better with GA than with the NDVI. Even so, the RGB_canopy_ indices also seemed to saturate for high GY but to a lesser extent than the NDVI because they mainly depend on changes in pigment concentration and the canopy LAI is less affected in the visible region than in the NIR region (Casadesus et al., 2007; Elazab et al., 2015).

In the case of the airborne NDVI data, the correlation with GY was also much lower than with GA taken on individual plots with GY. In fact, the images from the ADC multispectral camera have around four-fold less resolution than current digital camera technology (3.2 vs. 12 MP in our study, respectively). Although many ADC images were employed to obtain mosaics of the entire field trials, the resolution obtained at the flight altitude generated pixels which were mixed between pure vegetation, shadows and soil components. Such effects were successfully separated in the imagery collected at the near-canopy level with the RGB camera due to the higher resolution obtained. Altogether, the NDVIaerial provides a much lower amount of information than the GA and other VIs derived from RGB images taken at the plot level.

In the case of the LCC, it correlated strongly and linearly with grain yield across fertilization levels (Figure 2). In fact the leaf chlorophyll meters calculate a spectral ratio of the leaf transmittance to the near-infrared and red bands and they were primarily developed to assess N fertilization levels (Fox et al., 1994; Markwell et al., 1995). LCC indirectly predicts GY when a wide range of N conditions are considered and this is probably due to the relationship between chlorophyll content, leaf N and yield (Argenta et al., 2004).

Concerning the applications in breeding, the determination coefficients within N levels across genotypic means (Table 4) support the strength of RGB_canopy_ indices as phenotyping parameters. Thus, these indices were able to indicate the most efficient genotypes in terms of grain yield within each N fertilization level, whereas the NDVI performed much worse as a phenotyping parameter. Although genetic variability in maize hybrids in response to low N doses is high (Wang et al, 1999; Zaman-Allah et al., 2015) it has been scarcely exploited by breeding programs since they mainly focus on breeding for maize performance under favorable conditions (Machado and Fernandes, 2001). In this sense, the proposed phenotyping parameters herein, based on the use of RGB images, can significantly contribute to selection of maize hybrids resilient to low N as well as being more responsive to increases in N fertilization. For its part, LCC was unrelated to genotypic GY variation at any of the N-levels tested (Table 4), and this is in agreement with previous reports in maize that have noted LCC as not always being significantly correlated with genotypic differences in GY (Gallais and Coque, 2005).

### Crop monitoring and phenotyping parameters for leaf N assessment

The importance of leaf N concentration for N management and breeding lies not only in its potential contribution to grain N (Gallais and Coque, 2005) but is also due to it being a component of the nitrogen uptake efficiency (Serret et al., 2008). Moreover, leaf N is an indicator of leaf photosynthetic capacity contributing to grain yield (Richards, 2000) as well as a key fodder trait (Van der Wal et al., 2000). Therefore, the estimation of leaf N concentration within a given N fertilization treatment may provide valuable information about the genotypic efficiency for the uptake of N.

Our study highlights the potential of RGB indices for precise crop N management and for phenotyping genotypic performance under a wide-range of N conditions. As widely reported, LCC proved to be a very good indicator of leaf N concentration across nitrogen fertilization levels, therefore enabling monitoring of N application (Hirel et al., 2007). However, LCC failed to be effective as a phenotyping parameter, especially at high N-fertilization levels (Table 5). In contrast, the RGB_leaf_ indices demonstrated that they were the best genotypic predictors for leaf N concentration in the 0 to 80 kg·ha^−1^ N range. Thus, RGB indices at the leaf level have the potential to inform breeding programs about tolerance to N-deficiency stress in maize. This is a helpful insight because selection experiments have shown that the maximum genetic advance for low N is achieved when selecting in such N conditions (Gallais and Coque, 2005).

By contrast, in the highest N-fertilization level (160 kg ha^−1^) the RGB_leaf_ indices and LCC were probably saturated because they did not correlate with variations in leaf N concentration. For its part, the NDVI_aerial_ had an irregular trend as it was significantly correlated to changes in leaf N concentration at three of the five N fertilization levels (0, 80, and 160 kg ha^−1^) and these correlations were especially strong in the high N levels. As discussed above, besides of some plot variability and soil exposure, the poorer performance of the NDVI_aerial_ may be mainly explained by the relatively poor spectral resolution at the single plot level of the multispectral aerial images. Even so, according to our results this approach seems efficient for its implementation in aerial platforms.

### Use of leaf analytical parameters for crop management and phenotyping

Besides the leaf N concentration discussed above, other leaf N parameters like the N concentration on an area basis (N/LA) and the C/N ratio were strongly associated with GY across N fertilization levels. In the case of the leaf δ^15^N, its value gradually decreased as the N application rate increased. This trend is due to the absorption of N from chemical fertilizers that are highly depleted in ^15^N, whereas in the low N treatments plants absorb the N available in the soil, which is usually ^15^N-enriched (Bateman et al., 2005; Masuka et al., 2012). However, the genotypic effect was not significant for δ^15^N, which does not support the use of this isotopic signature for maize phenotyping under low N stress. These results disagree with previous studies in wheat where genotypic differences were found under N stress conditions (Araus et al., 2013).

In agreement with previous studies (Dercon et al., 2006), low N induced higher δ^13^C in maize, whereas it decreased in the high N fertilization treatments. This pattern of response appears related to the occurrence of some degree of water stress associated with a larger transpiring area due to nitrogen fertilization. In agreement with previous studies in maize, genotypic differences in leaf δ^13^C may be attributed to differences in transpiration efficiency, but the variation in δ^13^C was unrelated to GY within treatments (Cabrera-Bosquet et al., 2009).

Previous studies noted the relevance of SLA for the compositional and ecophysiological characterization of plants (Reich et al., 1998; Nautiyal et al., 2002). Several authors (Poorter and Evans, 1998; Meziane and Shipley, 2001) have reported a positive relationship between leaf N and SLA (Table 2). In turn, changes in SLA may be due to variations in leaf thickness and/or leaf density (Witkowski and Lamont, 1991). Increasing leaf density in low N conditions may be attributed to the increased synthesis of dense tissues such as sclerenchyma and vascular tissues that are rich in nitrogen-free substances (Garnier et al., 1997), whereas leaf thickness seems to have a minor role (Arendonk and Poorter, 1994). However, concerning its phenotyping use, SLA was shown to be homogeneous among the studied maize hybrids and unrelated to GY, as well as within a given N fertilization level, which excludes SLA as a phenotyping trait.

Regarding the relationship between VIs and the C/N ratio, most of the RGB indices (at the canopy and leaf levels) and both NDVI approaches were demonstrated as being even better correlated to the leaf C/N ratio than to leaf N concentration (Table S2). This finding may have considerable economic implications as the C/N ratio informs not only about the crop N status but also about the aerial biomass quality, including digestibility and nutritional quality (Van der Wal et al., 2000). Finally, all VIs and the LCC were better at capturing the differences in leaf N concentration than the amount of N concentration per unit leaf area (Table S2), thus avoiding the effect of leaf thickness or density. This evidence is enhanced by the weak relationship of the digital and spectral indices to SLA. This finding is particularly interesting in the case of LCC (SPAD readings), which has been previously positively correlated with leaf thickness and negatively correlated with SLA in other species (Marenco et al., 2009).

### Implications for breeding and crop management

The tested vegetation indices based on RGB images and to a lesser extent the NDVI demonstrated a high-throughput for the accurate prediction of several traits that are highly valuable for maize breeders and agronomists such as grain yield, leaf N concentration and the ratio of carbon to nitrogen under a wide range of N fertilization levels. Proper N fertilization management may be assisted considerably by using these parameters as decision criteria controlling the expected production and the uptake of N by the above-ground biomass. Beyond this, maize breeding programs may benefit from these findings through their application during the characterization of genotypic performance within N fertilization levels. In this way the selection of the most efficient genotypes in terms of grain production and/or N uptake may respond to the needs of low N stress tolerant maize varieties.

Vegetation indices derived from RGB images proved to be broad-use because they were previously employed satisfactorily in other crops under biotic and water stress conditions (Casadesus et al., 2007; Vergara-Diaz et al., 2015). Therefore, since this technique has proven its efficiency for the evaluation of plant growth and leaf color, it may be probably applicable to a wide range of biotic and abiotic stresses and crop species. Moreover our study also supports the use of this technique to assess genotypic differences in grain yield under good agronomical conditions.

Although the performance of the RGB indices (obtained from JPEG images) worked well in this study, future research may address the possibility of further improve their accuracy by using input images saved in a lossless compression format as TIFF or PNG. Despite of storage inconvenient, their larger capability (16 bit per pixel instead of 8 bit) may maintain higher quality detail from the visible spectrum. Another important consideration is the effect of changing light conditions when making these outdoor measurements. Despite of the good strength and repeatability of the results (Table S1) fluctuating ambient lighting should be considered as a possible source of error. Further research should also be targeted toward implementation and evaluation of similar RGB phenotyping methods in remotely piloted aerial platforms (Elazab et al., 2016; Rasmussen et al., 2016).

## Author contributions

BP, JC, MZ, and BM managed and directed the maize programme in the Southern Africa regional office of CIMMYT in Harare, Zimbabwe. MZ, PZ, and AH carried out the UAV flights for the obtainment of aerial measurements. On the other hand, JA, BM, JC, MZ, and OV conducted the field measurements and the collection of samples. AH and PZ processed the aerial images. OV analyzed the samples and other data and wrote the paper under the supervision of JA and with contributions from all the other authors.

### Conflict of interest statement

The authors declare that the research was conducted in the absence of any commercial or financial relationships that could be construed as a potential conflict of interest.

## References

[B1] Andrade-Sanchez P. Gore M. A. Heun J. T. Thorp K. R. Carmo-Silva A. E. French A. N. (2014). Development and evaluation of a field-based high-throughput phenotyping platform. Funct. Plant Biol. 41, 68–79. 10.1071/FP13126 32480967

[B2] Araus J. L. Cabrera-Bosquet L. Serret M. D. Bort J. Nieto-Taladriz M. T. (2013). Comparative performance of δ13C, δ18O and δ15N for phenotyping durum wheat adaptation to a dryland environment. Funct. Plant Biol. 40, 595–608. 10.1071/FP12254 32481133

[B3] Araus J. L. Cairns J. E. (2014). Field high-throughput phenotyping: the new crop breeding frontier. Trends Plant Sci. 19, 52–61. 10.1016/j.tplants.2013.09.008 24139902

[B4] Araus J. L. Sánchez C. Cabrera-Bosquet L. (2010). Is heterosis in maize mediated through better water use? New Phytol. 187, 392–406. 10.1111/j.1469-8137.2010.03276.x 20456048

[B5] Araus J. L. Slafer G. A. Royo C. Serret M. D. (2008). Breeding for yield potential and stress adaptation in cereals. Crit. Rev. Plant Sci. 27, 377–412. 10.1080/07352680802467736

[B6] Arendonk J. J. C. M. Poorter H. (1994). The chemical composition and anatomical structure of leaves of grass species differing in relative growth rate. Plant Cell Environ. 17, 963–970. 10.1111/j.1365-3040.1994.tb00325.x

[B7] Argenta G. Silva P. R. F. D. Sangoi L. (2004). Leaf relative chlorophyll content as an indicator parameter to predict nitrogen fertilization in maize. Cienc. Rural. 34, 1379–1387. 10.1590/S0103-84782004000500009

[B8] Barnes E. M. Clarke T. R. Richards S. E. Colaizzi P. D. Haberland J. Kostrzewski M. (2000). Coincident detection of crop water stress, nitrogen status and canopy density using ground based multispectral data, in *Proceedings of the 5th International Conference on Precision Agriculture* (Bloomington, MN), 16–19.

[B9] Bateman A. S. Kelly S. D. Jickells T. D. (2005). Nitrogen isotope relationships between crops and fertilizer: implications for using nitrogen isotope analysis as an indicator of agricultural regime. J. Agr Food Chem. 53, 5760–5765. 10.1021/jf050374h 15998145

[B10] Boegh E. Søgaard H. Broge N. Hasager C. B. Jensen N. O. Schelde K. (2002). Airborne multispectral data for quantifying leaf area index, nitrogen concentration, and photosynthetic efficiency in agriculture. Remote Sens. Environ. 81, 179–193. 10.1016/S0034-4257(01)00342-X

[B11] Buerkert A. Bationo A. Piepho H. P. (2001). Efficient phosphorus application strategies for increased crop production in sub-Saharan West Africa. Field Crop Res. 72, 1–15. 10.1016/s0378-4290(01)00166-6

[B12] Cabrera-Bosquet L. Sánchez C. Araus J. L. (2009). How yield relates to ash content, Δ13C and Δ18O in maize grown under different water regimes. Ann. Bot. 104, 1207–1216. 10.1093/aob/mcp229 19773272 PMC2766211

[B13] Cairns J. E. Hellin J. Sonder K. Araus J. L. MacRobert J. F. Thierfelder C. (2013). Adapting maize production to climate change in sub-Saharan Africa. Food Sec. 5, 345–360. 10.1007/s12571-013-0256-x

[B14] Cairns J. E. Sonder K. Zaidi P. H. Verhulst N. Mahuku G. Babu R. (2012). Maize production in a changing climate: impacts, adaptation and mitigation strategies. Adv. Agron. 144, 1–58. 10.1016/B978-0-12-394275-3.00006-7

[B15] Casadesus J. Kaya Y. Bort J. Nachit M. M. Araus J. L. Amor S. (2007). Using vegetation indices derived from conventional digital cameras as selection criteria for wheat breeding in water−limited environments. Ann. Appl. Biol. 150, 227–236. 10.1111/j.1744-7348.2007.00116.x

[B16] Casadesús J. Villegas D. (2014). Conventional digital cameras as a tool for assessing leaf area index and biomass for cereal breeding. J. Integr. Plant Biol. 56, 7–14. 10.1111/jipb.12117 24330531

[B17] Chen C. Pan J. Lam S. K. (2014). A review of precision fertilization research. Environ. Earth Sci. 71, 4073–4080. 10.1007/s12665-013-2792-2

[B18] Delgado J. A. Follett R. F. Buchleiter G. Stuebe A. Sparks R. T. Dillon M. A. (2001). Use of geospatial information for N management and conservation of underground water quality, in *Proceedings of the 3rd International Conference on Geospatial Information in Agriculture and Forestry* (Denver, CO), 5–7

[B19] Dercon G. Clymans E. Diels J. Merckx R. Deckers J. (2006). Differential ^13^C isotopic discrimination in maize at varying water stress and at low to high nitrogen availability. Plant Soil. 282, 313–326. 10.1007/s11104-006-0001-8

[B20] Elazab A. Bort J. Zhou B. Serret M. D. Nieto-Taladriz M. T. Araus J. L. (2015). The combined use of vegetation indices and stable isotopes to predict durum wheat grain yield under contrasting water conditions. Agr. Water Manage. 158, 196–208. 10.1016/j.agwat.2015.05.003

[B21] Elazab A. Ordóñez R. A. Savin R. Slafer G. A. Araus J. L. (2016). Detecting interactive effects of N fertilization and heat stress on maize productivity by remote sensing techniques. Eur. J. Agron. 73, 11–24. 10.1016/j.eja.2015.11.010

[B22] Evans R. D. (2001). Physiological mechanisms influencing plant nitrogen isotope composition. Trends Plant Sci. 6, 121–126. 10.1016/S1360-1385(01)01889-1 11239611

[B23] FAO (2013). Food and Agriculture Organisation of the United Nations; Statistic Division. Available online at: http://faostat.fao.org/

[B24] Feng Y. L. Fu G. L. Zheng Y. L. (2008). Specific leaf area relates to the differences in leaf construction cost, photosynthesis, nitrogen allocation, and use efficiencies between invasive and noninvasive alien congeners. Planta 228, 383–390. 10.1007/s00425-008-0732-2 18392694

[B25] Fischer T. Byerlee D. Edmeades G. (2014). Crop Yields and Global Food Security. Canberra, NSW: Australian Centre for International Agricultural Research, Monograph no. 158.

[B26] Fox R. H. Piekielek W. P. Macneal K. M. (1994). Using a chlorophyll meter to predict nitrogen fertilizer needs of winter wheat. Commun. Soil Sci. Plan. 25, 171–181. 10.1080/00103629409369027

[B27] Furbank R. T. Tester M. (2011). Phenomics–technologies to relieve the phenotyping bottleneck. Trends Plant Sci. 16, 635–644. 10.1016/j.tplants.2011.09.005 22074787

[B28] Gallais A. Coque M. (2005). Genetic variation and selection for nitrogen use efficiency in maize: a synthesis. Maydica 50, 531–547.

[B29] Garnier E. Cordonnier P. Guillerm J. L. Sonié L. (1997). Specific leaf area and leaf nitrogen concentration in annual and perennial grass species growing in Mediterranean old-fields. Oecologia 111, 490–498. 10.1007/s004420050262 28308109

[B30] Gitelson A. A. Kaufman Y. J. Stark R. Rundquist D. (2002). Novel algorithms for remote estimation of vegetation fraction. Remote Sens. Environ. 80, 76–87. 10.1016/S0034-4257(01)00289-9

[B31] Hasegawa K. Matsuyama H. Tsuzuki H. Sweda T. (2010). Improving the estimation of leaf area index by using remotely sensed NDVI with BRDF signatures. Remote Sens. Environ. 114, 514–519. 10.1016/j.rse.2009.10.005

[B32] Hatfield J. L. (2000). Precision Agriculture and Environmental Quality: Challenges for Research and Education. Ames, IA: USDA National Resources Conservation Service.

[B33] Hergert G. Ferguson R. Gotway C. Peterson T. (1996). The Impact of VRT-N application on N use efficiency of furrow irrigated corn, in Proceedings of the 3rd International Conference on Precision Agriculture, eds Robert P. C. Rust R. H. Larson W. E. (Madison, WI), 389–397.

[B34] Hirel B. Le Gouis J. Ney B. Gallais A. (2007). The challenge of improving nitrogen use efficiency in crop plants: towards a more central role for genetic variability and quantitative genetics within integrated approaches. J. Exp. Bot. 58, 2369–2387. 10.1093/jxb/erm097 17556767

[B35] Hobbs T. J. (1995). The use of NOAA-AVHRR NDVI data to assess herbage production in the arid rangelands of Central Australia. Int. J. Remote Sens. 16, 1289–1302. 10.1080/01431169508954477

[B36] Liebisch F. Kirchgessner N. Schneider D. Walter A. Hund A. (2015). Remote, aerial phenotyping of maize traits with a mobile multi-sensor approach. Plant Methods 11, 9. 10.1186/s13007-015-0048-8 25793008 PMC4365514

[B37] Machado A. T. Fernandes M. S. (2001). Participatory maize breeding for low nitrogen tolerance. Euphytica 122, 567–573. 10.1023/A:1017543426136

[B38] Marenco R. A. Antezana-Vera S. A. Nascimento H. C. S. (2009). Relationship between specific leaf area, leaf thickness, leaf water content and SPAD-502 readings in six Amazonian tree species. Photosynthetica 47, 184–190. 10.1007/s11099-009-0031-6

[B39] Markwell J. Osterman J. C. Mitchell J. L. (1995). Calibration of the Minolta SPAD-502 leaf chlorophyll meter. Photosynth. Res. 46, 467–472. 10.1007/BF00032301 24301641

[B40] Masuka B. Araus J. L. Das B. Sonder K. Cairns J. E. (2012). Phenotyping for abiotic stress tolerance in maize. J. Integr. Plant Biol. 54, 238–249. 10.1111/j.1744-7909.2012.01118.x 22443263

[B41] Meziane D. Shipley B. (2001). Direct and indirect relationships between specific leaf area, leaf nitrogen and leaf gas exchange. Effects of irradiance and nutrient supply. Ann. Bot. 88, 915–927. 10.1006/anbo.2001.1536

[B42] Monneveux P. Sheshshayee M. S. Akhter J. Ribaut J. M. (2007). Using carbon isotope discrimination to select maize (*Zea mays* L.) inbred lines and hybrids for drought tolerance. Plant Sci. 173, 390–396. 10.1016/j.plantsci.2007.06.003

[B43] Morgounov A. Gummadov N. Belen S. Kaya Y. Keser M. Mursalova J. (2014). Association of digital photo parameters and NDVI with winter wheat grain yield in variable environments. Turk. J. Agric. For. 38, 624–632. 10.3906/tar-1312-90

[B44] Nautiyal P. C. Rachaputi N. R. Joshi Y. C. (2002). Moisture-deficit-induced changes in leaf-water content, leaf carbon exchange rate and biomass production in groundnut cultivars differing in specific leaf area. Field Crop Res. 74, 67–79. 10.1016/S0378-4290(01)00199-X

[B45] Petropoulos G. P. Kalaitzidisz C. (2012). Multispectral vegetation indices in remote sensing: an overview. Ecol. Model 2, 15–39.

[B46] Poorter H. Evans J. R. (1998). Photosynthetic nitrogen-use efficiency of species that differ inherently in specific leaf area. Oecologia 116, 26–37. 10.1007/s004420050560 28308535

[B47] Rasmussen J. Ntakos G. Nielsen J. Svensgaard J. Poulsen R. N. Christensen S. (2016). Are vegetation indices derived from consumer-grade cameras mounted on UAVs sufficiently reliable for assessing experimental plots? Eur. J. Agron. 74, 75–92. 10.1016/j.eja.2015.11.026

[B48] Reich P. B. Ellsworth D. S. Walters M. B. (1998). Leaf structure (specific leaf area) modulates photosynthesis–nitrogen relations: evidence from within and across species and functional groups. Funct. Ecol. 12, 948–958. 10.1046/j.1365-2435.1998.00274.x

[B49] Richards R. A. (2000). Selectable traits to increase crop photosynthesis and yield of grain crops. J. Exp. Bot. 51, 447–458. 10.1093/jexbot/51.suppl_1.447 10938853

[B50] Roberts R. English B. Mahajanashetti S. (2001). Environmental and economic effects of spatial variability and weather, in Proceedings of the 3rd European Conference on Precision Agriculture, eds Blackmore S. Grenier G. (Montpellier), 545–550.

[B51] Rorie R. L. Purcell L. C. Karcher D. E. King C. A. (2011). The assessment of leaf nitrogen in corn from digital images. Crop Sci. 51, 2174–2180. 10.2135/cropsci2010.12.0699

[B52] Serret M. D. Ortiz Monasterio I. Pardo A. Araus J. L. (2008). The effects of urea fertilisation and genotype on yield, nitrogen use efficiency, δ^15^N and δ^13^C in wheat. Ann. App. Biol. 153, 243–257. 10.1111/j.1744-7348.2008.00259.x

[B53] Seul M. O'Gorman L. Sammon M. J. (2000). Practical Algorithms for Image Analysis. *Description, Examples, and Code* Cambridge, UK; Cambridge University Press

[B54] Stewart W. M. Dibb D. W. Johnston A. E. Smyth T. J. (2005). The contribution of commercial fertilizer nutrients to food production. Agron. J. 97, 1–6. 10.2134/agronj2005.0001

[B55] Svensgaard J. Roitsch T. Christensen S. (2014). Development of a mobile multispectral imaging platform for precise field phenotyping. Agronomy 4, 322–336. 10.3390/agronomy4030322

[B56] Trussell H. J. Vrhel M. Saber E. (2005). Color image processing. IEEE Signal Proc. Mag. 22, 14–22. 10.1109/MSP.2005.1407711

[B57] Van der Wal R. Madan N. Van Lieshout S. Dormann C. Langvatn R. Albon S. D. (2000). Trading forage quality for quantity? Plant phenology and patch choice by Svalbard reindeer. Oecologia 123, 108-115. 10.1007/s004420050995 28308735

[B58] Vergara-Diaz O. Kefauver S. C. Elazab A. Nieto-Taladriz M. T. Araus J. L. (2015). Grain yield losses in yellow-rusted durum wheat estimated using digital and conventional parameters under field conditions. Crop J. 3, 200–210. 10.1016/j.cj.2015.03.003

[B59] Wang F. Fraisse C. W. Kitchen N. R. Sudduth K. A. (2003). Site-specific evaluation of the CROPGRO-soybean model on Missouri claypan soils. Agr. Syst. 76, 985–1005. 10.1016/S0308-521X(02)00029-X

[B60] Wang R. L. Stec A. Hey J. Lukens L. Doebley J. (1999). The limits of selection during maize domestication. Nature 398, 236–239. 10.1038/18435 10094045

[B61] Wezel A. Casagrande M. Celette F. Vian J. F. Ferrer A. Peigné J. (2014). Agroecological practices for sustainable agriculture. A review. Agron. Sustain. Dev. 34, 1–20. 10.1007/s13593-013-0180-7

[B62] White J. W. Andrade-Sanchez P. Gore M. A. Bronson K. F. Coffelt T. A. Conley M. M. (2012). Field-based phenomics for plant genetics research. Field Crop Res. 133, 101–112. 10.1016/j.fcr.2012.04.003

[B63] Witkowski E. T. F. Lamont B. B. (1991). Leaf specific mass confounds leaf density and thickness. Oecologia 88, 486–493. 10.1007/BF00317710 28312617

[B64] Zaman-Allah M. Vergara O. Araus J. L. Tarekegne A. Magorokosho C. Zarco-Tejada P. J.. (2015). Unmanned aerial platform-based multi-spectral imaging for field phenotyping of maize. Plant Methods 11:35. 10.1186/s13007-015-0078-2 26106438 PMC4477614

[B65] Zhou B. Elazab A. Bort J. Vergara O. Serret M. D. Araus J. L. (2015). Low-cost assessment of wheat resistance to yellow rust through conventional RGB images. Comput. Electron. Agr. 116, 20–29. 10.1016/j.compag.2015.05.017

