# Supplementary material for: A Novel Remote Sensing Approach for Prediction of Maize Yield Under Different Conditions of Nitrogen Fertilization
Source: Front Plant Sci. 2016 May 18;7:666. doi: 10.3389/fpls.2016.00666 (PMC4870241; doi:10.3389/fpls.2016.00666)
Supplement: Supplementary file 1 [file DataSheet1.DOCX]

Supplementary Material

**A novel remote sensing approach for prediction of maize yield under different conditions of nitrogen fertilization**

**Omar Vergara-Díaz^1^, Mainassara Zaman-Allah^2^, Benhildah Masuka^2^, Alberto Hornero^3^, Pablo Zarco-Tejada^3^, Boddupalli Prasanna^2^, Jill E. Cairns^2^, José Luís Araus^1*^**

*** Correspondence:** Prof. José Luís Araus, Unit of Plant Physiology, Department of Plant Biology, Faculty of Biology, University of Barcelona, 643 Diagonal, Barcelona, 08028, Spain.

# Supplementary Tables

**Table S1**. Determination coefficients (R^2^) of RGB-indices from canopy RGB images explaining the variation between replicated plot images (n = 57). Indices were calculated from images taken in the same set of plots in a sunny and a cloudy days nearly consecutive.

|  |  | R^2^ | p-value |
| --- | --- | --- | --- |
| RGB_canopy_ indices | |  |  |
|  | hue | 0.437 | <0.001 |
|  | a* | 0.716 | <0.001 |
|  | b* | 0.597 | <0.001 |
|  | u* | 0.722 | <0.001 |
|  | v* | 0.541 | <0.001 |
|  | GA | 0.895 | <0.001 |
|  | GGA | 0.795 | <0.001 |

**Table S2**. Pearson correlation coefficients between leaf and canopy RGB indices, NDVI at ground and aerial levels, leaf chlorophyll content (LCC) and grain yield (GY) with nitrogen per unit area (N/area), the carbon to nitrogen ratio (C/N), nitrogen isotope composition (δ^15^N), carbon isotope composition (δ^13^C), specific leaf area (SLA) and leaf nitrogen concentration (%N). *, P < 0.05; **, P < 0.001; ns, non-significant.

|  |  | N/LA | C/N | δ^15^N | δ^13^C | SLA | %N |
| --- | --- | --- | --- | --- | --- | --- | --- |
| RGB_leaf_ indices | |  |  |  |  |  |  |
|  | hue | -0.451 ** | 0.503 ** | 0.464 ** | 0.278 * | -0.068 ns | -0.540 ** |
|  | a* | 0.718 ** | -0.854 ** | -0.505 ** | -0.566 ** | 0.120 ns | 0.831 ** |
|  | b* | -0.645 ** | 0.826 ** | 0.485** | 0.579 ** | -0.193 ns | -0.788 ** |
|  | u* | 0.725 ** | -0.843 ** | -0.511 ** | -0.542 ** | 0.093 ns | 0.826 ** |
|  | v* | -0.621 ** | 0.843 ** | 0.553 ** | 0.589 ** | -0.259* | -0.802 ** |
|  | GA | -0.005 ns | -0.243 * | -0.418 ** | -0.187 ns | 0.373 ** | 0.220 * |
|  | GAA | -0.107 ns | -0.184 ns | -0.377 ** | -0.047 ns | 0.450** | 0.159 ns |
| RGB_canopy_ indices | |  |  |  |  |  |  |
|  | hue | 0.362 ** | -0.462 ** | -0.313 * | -0.028 ns | 0.098 ns | 0.430 ** |
|  | a* | 0.000 ns | 0.096 ns | 0.092 ns | -0.215 * | -0.076 ns | 0.032 ns |
|  | b* | -0.058 ns | 0.010 ns | -0.076 ns | -0.022 ns | 0.047 ns | -0.030 ns |
|  | u* | -0.174 ns | 0.301 * | -0.248 * | 0.101 ns | -0.124 ns | -0.245 * |
|  | v* | -0.518 ** | 0.604 ** | 0.486 ** | 0.361 ** | -0.142 ns | -0.623 ** |
|  | GA | 0.397 ** | -0.558 ** | -0.562 ** | -0.124 ns | 0.181 ns | 0.529 ** |
|  | GAA | 0.398 ** | -0.574 ** | -0.527 ** | -0.118 ns | 0.202 * | 0.533 ** |
| Spectral Indices | |  |  |  |  |  |  |
|  | NDVI_aerial_ | 0.221 * | -0.495 ** | -0.672 ** | -0.190 ns | 0.298 * | 0.477 ** |
|  | NDVI_ground_ | 0.271 * | -0.364 ** | -0.350 ** | 0.043 ns | 0.199 * | 0.341 ** |
| LCC | | 0.748 ** | -0.898** | -0.678 ** | -0.464 ** | 0.195 ns | 0.905 ** |
| GY | | 0.608 ** | -0.676 ** | -0.555 ** | -0.249 * | 0.102 ns | 0.687 ** |
| %N | | 0.816 ** | -0.967 ** | -0.580 ** | -0.590 ** | 0.220 * | 1 |
